# Genome-wide characterization of the *WAK* gene family and expression analysis under plant hormone treatment in cotton

**DOI:** 10.1186/s12864-021-07378-8

**Published:** 2021-01-28

**Authors:** Lingling Dou, Zhifang Li, Qian Shen, Huiran Shi, Huaizhu Li, Wenbo Wang, Changsong Zou, Haihong Shang, Hongbin Li, Guanghui Xiao

**Affiliations:** 1 School of Chemistry and Chemical Engineering, Xianyang Normal University, Xianyang, 712000 Shaanxi China; 2 State Key Laboratory of Cotton Biology, State Key Laboratory of Crop Stress Adaptation and Improvement, School of Life Sciences, Henan University, 85 Minglun Street, Kaifeng, 475001 Henan China; 3 State Key Laboratory of Cotton Biology, Institute of Cotton Research, Chinese Academy of Agricultural Sciences, Anyang, 455000 Henan China; 4 College of Life Sciences, Shaanxi Normal University, Xi’an, 710119 China; 5 Zhengzhou Research Base, State Key Laboratory of Cotton Biology, Zhengzhou University, Zhengzhou, China; 6 College of Life Sciences, Key Laboratory of Xinjiang Phytomedicine Resource and Utilization of Ministry of Education, Shihezi University, Shihezi, 832003 China

**Keywords:** *Gossypium hirsutum*, *WAK* genes, Expression analysis

## Abstract

**Background:**

Wall-associated kinases (WAK), one of the receptor-like kinases (RLK), function directly in the connection and communication between the plant cell wall and the cytoplasm. *WAK* genes are highly conserved and have been identified in plants, such as rice, but there is little research on the *WAK* gene family in cotton.

**Results:**

In the present study, we identified 29 *GhWAK* genes in *Gossypium hirsutum*. Phylogenetic analysis showed that cotton WAK proteins can be divided into five clades. The results of synteny and Ka/Ks analysis showed that the *GhWAK* genes mainly originated from whole genome duplication (WGD) and were then mainly under purifying selection. Transcriptome data and real-time PCR showed that 97% of *GhWAK* genes highly expressed in cotton fibers and ovules. β-glucuronidase (GUS) staining assays showed that *GhWAK5* and *GhWAK16* expressed in Arabidopsis leaf trichomes. Fourteen *GhWAK* genes were found to possess putative gibberellin (GA) response elements in the promoter regions, 13 of which were significantly induced by GA treatment. Ten *GhWAK* genes contained auxin (IAA) response elements and the expression level of nine *GhWAKs* significantly increased under auxin treatment.

**Conclusions:**

We provide a preliminary analysis of the *WAK* gene family in *G. hirsutum*, which sheds light on the potantial roles of *GhWAK* genes in cotton fiber cell development. Our data also provides a useful resource for future studies on the functional roles of *GhWAK* genes.

**Supplementary Information:**

The online version contains supplementary material available at 10.1186/s12864-021-07378-8.

## Background

The cell wall is a slightly elastic structure surrounding the cell membrane and is a complex network composed of cellulose, hemicellulose, pectin and a small amount of structural proteins [1]. The cell wall functions in cell morphological structure and is the first defense against pathogen invasion [2]. Receptor like kinases (RLKs), are a kind of important plant protein kinases. A typical RLK protein contains a putative extracellular domain (ECD), a hydrophobic transmembrane region and a cytoplasmic Ser/Thr kinase domain [3], which could be activated by external and internal stimulus and play important roles in plant growth and development. Studies have shown that the wall-associated kinases (WAKs) and the proline rich extensine-like receptor kinases (PERKs) function in signal transduction between the extracellular matrix and cytoplasm [4].

WAKs are linked with the cell wall and are important proteins in the connection between plant cell wall and cytoplasm. Typical WAK structures mainly consist of four domains, including ECD, transmembrane domain, epidermal growth factor-like (EGF) and intracellular kinase domain [5]. The intracellular domain of WAKs is composed of Ser/Thr kinase, which functions in intracellular signal transduction [6]. Protease treatments demonstrated that the ECD locates at the N-terminal, is a galacturonan-binding extracellular domain, which directly connected to the cell wall [7, 8]. Near the transmembrane domain, there are EGF-repeats containing 12 conserved cysteine residues [5]; these amino acid residues are thought to be directly involved in protein-protein interactions and are related to protein functions. The transmembrane structure of WAK indicates that WAK is essential in signaling between the cytoplasm and the cell wall [9]. Results of enzyme-linked immunosorbent assays (ELISAs) and pectinase treatment suggest that WAKs covalently bind to pectin [9, 10], which is the main component of the cell wall and is involved in cell elongation and pathogen resistance.

The first member of the *WAK* gene family, *pro25*, was found in *Arabidopsis*, and contains the Ser/Thr kinase domain. After analyzing its biochemical characteristics, pro25 was found to be closely linked to the cell wall, so they renamed it WAK1 (wall-associated kinase 1) and proposed the concept of cell wall-linked protein kinase [3]. Researchers then discovered a family of genes containing *WAK1*, *WAK2*, *WAK3*, *WAK4* and *WAK5* in *Arabidopsis* and they were highly conservative [7].

WAKs play important functions in cell elongation. In *Arabidopsis*, *AtWAK4* antisense gene resulted in significantly decreased cell elongation and inhibited lateral root development [11]. Also, antisense plants of five members of the *WAK* family in *Arabidopsis* showed smaller leaves than control plants with shorter cells [9, 12]. The *WAK2* null allele, *wak2–1*, caused a loss of cell expansion in roots. In addition, *WAK2* affected cell elongation by regulating the expression level of a vacuolar invertase, which separates sucrose into glucose and fructose to increase the concentration of solute and promote cell water swelling, elevating intracellular turgor and resulting in a loose cell wall [1]. Expression analysis of the five *AtWAK* members also showed that *WAK*s expressed at organ junctions, shoot apical meristems, root apical meristems, and expanding leaves [9]. *WAK*s expressed throughout plant development and are required for cell expansion and elongation. Plant cell expansion and elongation mechanisms are very complicated and involve phytohormones, such as gibberellin (GA) and auxin (IAA) [13–16].

In recent years, studies have shown that WAKs are pectin receptors not only required for elongation but also for an induced stress response [17]. In *Arabidopsis*, *WAK1* can be induced by pathogen infection or by exogenous salicylic acid (SA) [18]. RLK family genes *AtWAK1–3* are involved in the activation of the secondary bile acid deoxycholic acid (DCA), which can activate the plant immune system to decrease two kinds of bacteria [19]. *ZmWAK* responds to the maize fungal diseases corn leaf blight and head smut disease [20, 21]. In rice, *OsWAK14*, *OsWAK91* and *OsWAK92* positively regulate quantitative resistance and *OsWAK112d* is a negative regulator of resistance to rice blast fungus *Magnaporthe oryzae* [22, 23]. Wheat *TtWAK2* enhances wheat resistance to *Fusarium graminearum* by binding to pectin fragments [24], and *OsWAK/Xa4* increases rice blight resistance by strengthening the cell wall through increasing cellulose synthesis [25]. *WAK* genes function in resistance to bacterial and fungal diseases in cereal plants with increasing biosynthesis of cellulose and phytoalexin to increase cell wall strength [20, 21, 25, 26]. WAK proteins are also essential in responding to plant abiotic stresses. *OsWAK11* defends against excess copper by regulating the methylesterification of the cell wall [27]. Overexpression of *AtWAK1* enhanced *A. thaliana* root tolerance to aluminum [28].

*G. hirsutum* is an allotetraploid species. The homeologous of chloroplast DNAs and the large subunit of ribulose bisphosphate carboxylase analysis among *Gossypium* species showed that the allotetraploid cotton species originated from the hybridization of female parent *G. arboreum* (AA) and male parent *G. raimondii* (DD) 1–2 million years ago [29, 30]. *G. hirsutum* is planted worldwide because its valuable fiber is used as raw material in the textile industry and the length and strength of its fibers are very important qualities for this industry [31, 32]. Cotton fibers start from ovule epidermal cells and rapidly elongate to form a single cell seed coat [33, 34]. Cotton fiber development is divided into four overlapping stages: initiation, elongation, secondary cell wall deposition, and maturation [35]. The fiber length is determined during its elongation stage, 0 to 26 days post-anthesis (DPA), to reach its final length [36]. The cellulose of the fiber cell wall is synthesized from 15 to 40 DPA, which determines the quality of fiber strength [37]. WAKs directly connected with the cell wall and functioned in cell elongation and cellulose synthesis, *WAK* genes is important for cotton fiber development and lays foundation for improving fiber qualities through over-expression or silence of *GhWAKs* by genetic engineering. In the present study, we performed whole-genome analysis on the *WAK* gene family in *G. hirsutum*. The potential function of the *WAK* gene family in cotton fiber development was further elucidated through phylogenetic relationships, chromosome positions, expression profiles, GUS staining and protein structure analysis.

## Results

### Identification of *WAK* genes in *Gossypium* species and chromosomal distribution analysis

**Fig. 1:**
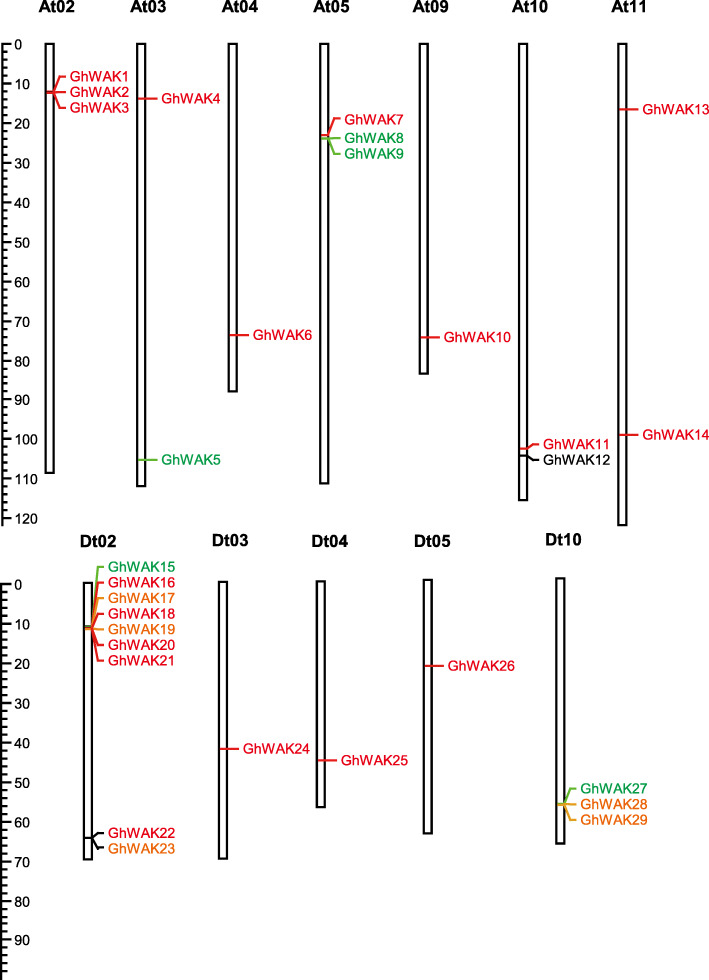
Chromosomal distribution of *GhWAK*s. Red lines show gene pairs involved in segmental duplication, green lines show gene pairs involved in tandem duplication, orange lines show gene pairs involved in proximal duplication

Whole genome sequences of three sequenced cotton species - *G. hirsutum* acc. TM-1 (ZJU_v2.1) [29], *G. arboreum* (CRI_v3.0) [38] and *G. raimondii* (JGI_v2.1) [39] - were used to identify WAK proteins. *Arabidopsis* AtWAK protein sequences were used as queries to search against the three reference genomes to screen out candidate WAK proteins in cotton. All the candidates (GhWAKs, GaWAKs and GrWAKs) with the four domains (signal peptide, 1–3 EGFs, transmembrane domain and protein kinase domain) were predicted to encode WAK proteins. In total, there were 29, 19 and 14 *WAK* genes in *G. hirsutum*, *G. arboreum* and *G. raimondii*, respectively. According to their relative chromosome locations, the 29 candidate *GhWAK* genes were named as *GhWAK1* to *GhWAK29* from chromosomes At01 to Dt13 (Fig. 1). Chromosome distribution analysis showed that the 29 *GhWAK*s unequally distributed on 12 chromosomes including seven At subgenomes and five Dt subgenomes. In chromosome Dt_02 nine *GhWAK* genes clustered together; At_02, At_05 and Dt_10 each had three *GhWAK* genes; At_10 had two *GhWAK* genes (Fig. 1); and all other chromosomes each had one *GhWAK*.

The number of amino acids (aa) in the GhWAK protein sequences ranged from 606 aa (GhWAK15) to 1200 aa (GhWAK29) with an average length of 755 aa. The molecular weights (MWs) of the predicted encoded proteins varied from 67.36 kDa (GhWAK15) to 134.02 kDa (GhWAK29) with an average of 84.32 kDa. According to the isoelectric point (pI) analysis, 20 GhWAKs had pI < 7.0 (with an average of 6.02) and were acidic in nature, whereas nine *GhWAK*s were predicted to encode proteins with pI > 7.0 (average of 8.34) and were basic in nature. Based on the instability index analysis, 23 GhWAK proteins have instability index values less than 40.0 and six GhWAK proteins have instability index values greater than 40.0 (GhWAK5, GhWAK15, GhWAK16, GhWAK20, GhWAK22 and GhWAK23). All the GhWAK proteins had negative grand average of hydropathicity (GRAVY) scores, indicating that all the GhWAK proteins were hydrophilic (Table 1). The detailed physicochemical parameters of GaWAK and GrWAK proteins were listed in Additional file 2: Table S1.

**Table 1:** Physicochemical parameters of 29 *GhWAK* genes in *G.hirsutum* genome

Gene ID	Gene Name	Chromosomes	Number of amino acids	Molecular weight	Theoretical pI	Instability index	Aliphatic index	Grand average of hydropathicity (GRAVY)
GH_A02G0756	GhWAK1	A02	751	83,410.25	6.17	35.88	87.88	−0.188
GH_A02G0757	GhWAK2	A02	743	82,937.57	5.91	39.16	85.25	−0.153
GH_A02G0759	GhWAK3	A02	706	78,946.34	6.8	31.85	85.58	−0.238
GH_A03G0713	GhWAK4	A03	650	72,826.38	8.56	35.71	85	−0.176
GH_A03G1857	GhWAK5	A03	1014	113,495.2	5.89	42.75	72.38	−0.371
GH_A04G1037	GhWAK6	A04	636	70,572.08	8.62	33.77	94.26	−0.051
GH_A05G2392	GhWAK7	A05	752	83,052.99	5.11	32.64	84.88	−0.216
GH_A05G2396	GhWAK8	A05	709	79,129.46	8.35	36.54	82.61	−0.201
GH_A05G2397	GhWAK9	A05	746	82,336.37	6.52	31.05	84.68	−0.213
GH_A09G1705	GhWAK10	A09	637	70,724.12	8.79	34.59	93.36	−0.103
GH_A10G1999	GhWAK11	A10	684	76,591.69	6.38	36.21	84.06	−0.214
GH_A10G2055	GhWAK12	A10	745	83,174.38	5.99	39.57	79.93	−0.236
GH_A11G1483	GhWAK13	A11	654	73,748.88	6.4	39.24	88.04	−0.193
GH_A11G2681	GhWAK14	A11	636	71,097.94	8.7	38.26	87.04	−0.148
GH_D02G0763	GhWAK15	D02	606	67,363.5	7.09	40.44	81.85	−0.23
GH_D02G0764	GhWAK16	D02	745	82,717.14	5.2	40.76	88.97	−0.154
GH_D02G0769	GhWAK17	D02	745	82,795.27	5.33	38.37	88.05	−0.157
GH_D02G0772	GhWAK18	D02	747	83,034.53	5.63	35.96	84.95	−0.207
GH_D02G0775	GhWAK19	D02	751	83,325.86	5.83	37.04	85.43	−0.222
GH_D02G0777	GhWAK20	D02	743	83,260.15	6.2	40.28	85.9	−0.156
GH_D02G0778	GhWAK21	D02	716	80,229	6.54	35.66	86.7	−0.209
GH_D02G2021	GhWAK22	D02	993	111,861.5	7.96	44.65	78.89	−0.337
GH_D02G2022	GhWAK23	D02	1037	116,248.6	6.14	45.02	74.06	−0.368
GH_D03G1229	GhWAK24	D03	691	76,829.83	8.55	33.01	84.91	−0.176
GH_D04G1370	GhWAK25	D04	636	70,615.13	8.45	33.79	95.33	−0.039
GH_D05G2414	GhWAK26	D05	753	83,958.06	5.23	35.97	83.73	−0.254
GH_D10G2159	GhWAK27	D10	725	80,672.86	6.73	34.01	80.54	−0.218
GH_D10G2163	GhWAK28	D10	769	86,285.9	6.24	34.86	77.44	−0.253
GH_D10G2171	GhWAK29	D10	1200	134,018.2	6.22	35.24	74.99	−0.255

### Gene structure and phylogenetic relationships among GhWAK proteins

**Fig. 2:**
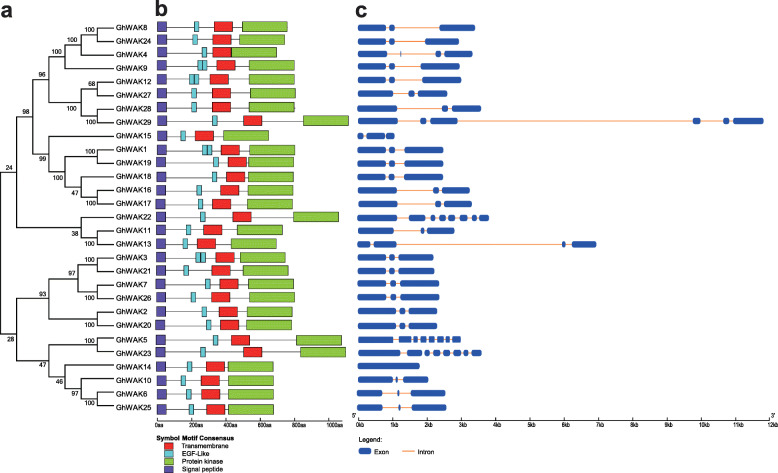
Phylogenetic relationships of protein architecture and gene structure. **a** Phylogenetic relationships of *GhWAK* genes. The CDS of *GhWAK*s were used to construct the phylogenetic tree in MEGA 7.0, using the NJ-method and tested with 1000 bootstrap iterations. **b** Conserved domains of GhWAK proteins. Four motifs of GhWAK proteins determined using MEME. The red rectangular box indicates the transmembrane domain, the blue rectangular box indicates the EGF-Like domain, the green rectangular box indicates proteins kinase, and the purple rectangular box indicates signal peptide. **c** Gene structure analysis of *GhWAK*s. The exons and introns are indicated with blue filled boxes and orange lines, respectively

To fully display the protein domains and gene structures, a phylogenetic tree was constructed with the coding sequences (CDS) of *GhWAK*s (Fig. 2a). According to previous studies, WAK proteins contain highly conserved domains. All the GhWAK proteins have a signal peptide at the N-terminal end, the EGF-like domain is located near the transmembrane domain and the protein kinase domain is located at the C-terminal end (Fig. 2b). The intron and exon structure diagrams of *GhWAK* genes were constructed according to the CDS of the genes. *GhWAK14* gene has no intron structure, *GhWAK13* has four exons, and most of the *GhWAK*s have three exons (Fig. 2c).

### Phylogenetic analysis of GhWAK proteins

**Fig. 3:**
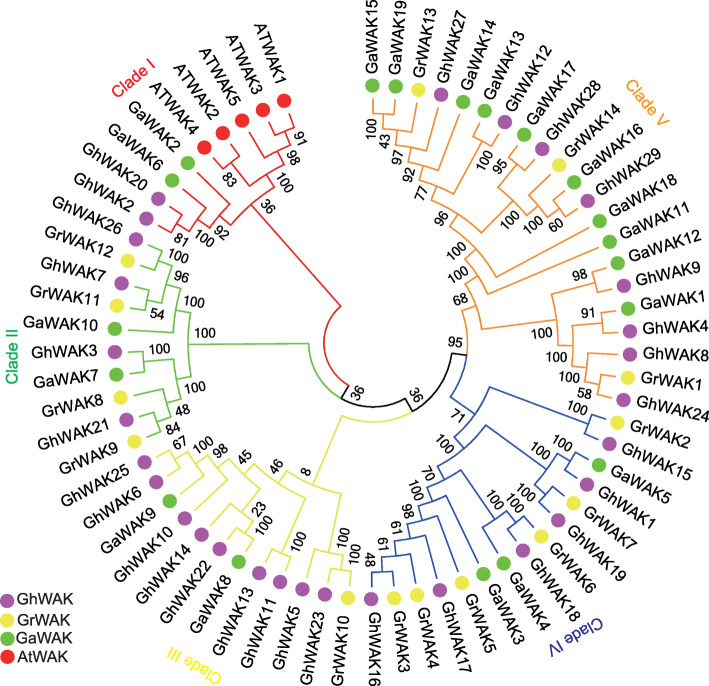
Phylogenetic tree of GhWAK GaWAK, GrWAK and AtWAK proteins. The phylogenetic tree was constructed using the full length of 29 GhWAKs, 19 GaWAKs, 14 GrWAKs and five AtWAKs, AtWAK1 (AT1G21210.1), AtWAK2 (AT1G21230.1), AtWAK3 (AT1G21240.1), AtWAK4 (AT1G21250.1) and AtWAK5 (AT1G21270.1) protein amino acid sequences. The phylogenetic tree was constructed using the neighbor-joining method with bootstrapping with 1000 iterations by MEGA 7.0

The phylogenetic tree of the *WAK* gene family contains *WAK* gene members from four species: *G. hirsutum*, *G. arboreum*, *G. raimondii* and *A. thaliana*. The phylogenetic tree was constructed using the neighbor-joining (NJ) method and tested by 1000 bootstrap replicates. The tree was resolved into five subgroups: Clade I, Clade II, Clade III, Clade IV and Clade V (Fig. 3). The *GhWAK* genes that clustered together in Fig. 1 were also preferentially clustered into the same subgroup in the phylogenetic tree. For example, *GhWAK2* and *GhWAK3* were clustered on chromosome At_02 and they were also clustered together into Clade II in the phylogenetic tree in Fig. 3. This phenomenon indicated that *GhWAK* genes may originate from gene duplication.

### Duplication events of *GhWAK*s

Gene duplication events are usually the main reason for expansion of gene family members. Therefore, we further analyzed the gene duplication types of the *WAK* gene family in *G. hirsutum*, *G. arboreum* and *G. raimondii* using MCScanX. In total, 18 *GhWAK*s (accounting for 62.07%, ten *GhWAK*s from subgenome_At and eight *GhWAKs* from subgenome_Dt) were produced by whole genome duplication (WGD), five *GhWAK*s (17.24%) were produced by tandem duplication and five *GhWAK*s (17.24%) were produced by proximal duplication event (Additional file 2: Table S2). These results indicate that WGD events play a predominant role in the expansion of *GhWAK* genes, while *GaWAK*s and *GrWAK*s were mainly produced from tandem duplication events, accounting for 57.89 and 42.86%, respectively (Additional file 2: Table S2).

**Fig. 4:**
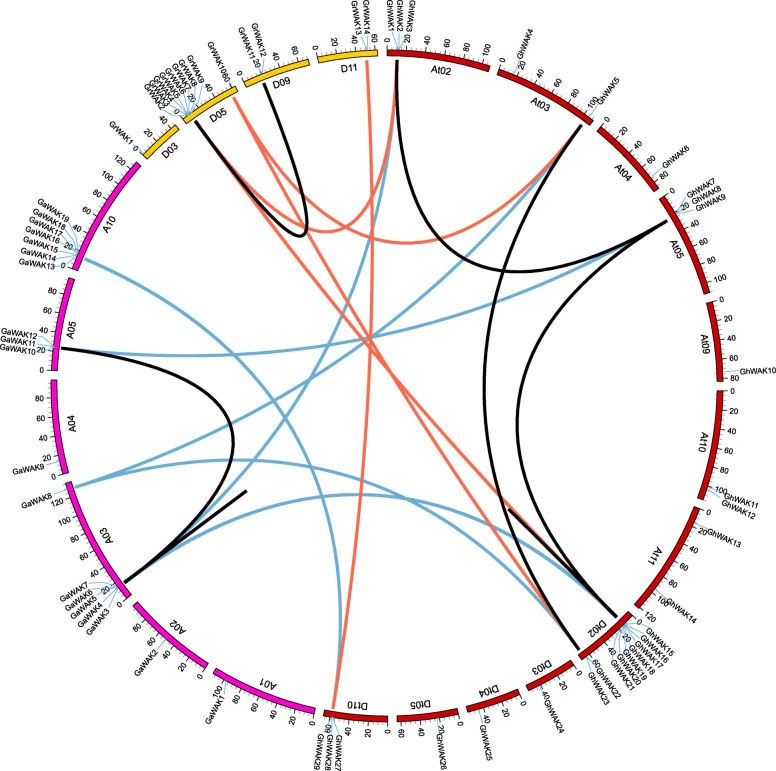
The synteny relationships of *WAK* genes among three sequenced cotton species. *G. hirsutum*, *G. raimondii* and *G. arboreum* chromosomes are indicated in red, orange and pink, respectively. The putative orthologous WAKs between *G. arboreum* and *G. hirsutum*, and between *G. raimondii* and *G. hirsutum* are connected by blue and orange lines, respectively. Black lines connect the putative paralogous gene pairs (*GhWAK15*/*GhWAK16*, *GhWAK2*/*GhWAK9*, *GhWAK8*/*GhWAK20*, *GhWAK5*/*GhWAK22*, *GaWAK7*/*GaWAK6*, *GaWAK3*/*GaWAK12* and *GrWAK7*/*GrWAK11*)

To compare the synteny and collinearity relationships of *WAK*s among *G. arboreum*, *G. raimondii* and *G. hirsutum*, we identified the orthologous and paralogous genes among the three released cotton genomes (Additional file 2: Table S3). Twelve *GhWAK*s had orthologous genes in *G. arboreum*, showing an AA genome origin, and seven *GhWAK*s genes had orthologous genes in *G. raimondii*, showing a DD genome origin (Fig. 4 and Additional file 1: Figure S1). To further analyze the gene duplication events of *GhWAK*s, we characterized four paralogous gene pairs (*GhWAK15*/*GhWAK16*, *GhWAK9*/*GhWAK2*, *GhWAK8*/*GhWAK20*, and *GhWAK5*/*GhWAK22*) in the *G. hirsutum* genome, two paralogous gene pairs (*GaWAK7*/*GaWAK6* and *GaWAK3*/*GaWAK12*) in *G. arboreum* and one paralogous gene pair (*GrWAK7*/*GrWAK11*) in *G. raimondii*. Homologous exchange was observed among cotton genomes, which may lead to the orthologous gene pairs in different numbered chromosome among *G. raimondii*, *G. arboreum* and *G. hirsutum*.

To further investigate the selective pressure after a gene duplication event, the non-synonymous (Ka) and synonymous (Ks) substitution rates were calculated. All the *GhWAK* gene pairs had Ka/Ks < 1.0 (Additional file 2: Table S4), indicating that the *GhWAK* genes had experienced purifying selection pressure after gene duplication events. Furthermore, the Ks ratio is less affected by selection and is usually used to estimate the evoluting divergence time [40]. The Ks ratios of six *GhWAK* gene pairs ranged from 0.0755 to 0.946, and duplication events occurred from approximately 14.51 million years ago (MYA) to 181.90 MYA (Additional file 2: Table S4). The Ks ratios of four *GaWAK* gene pairs ranged from 0.204 to 0.680, and the tandem duplication events occurred 39.20 MYA to 130.81 MYA. The Ks ratios of three *GrWAK* gene pairs ranged from 0.0362 to 0.272, and the tandem and proximal duplication events occurred 6.97 MYA to 52.23 MYA. These results suggest that the duplication events occurred before the polyploidization event because the *G. hirsutum* polyploidization occurred about 1–2 MYA.

### Expression patterns of *GhWAKs* in different tissues

To investigate the tissue-specific expression patterns of *GhWAK* gene family members, transcriptome data from the roots, stems, leaves and 0 dpa ovules were used. As shown in Additional file 1: Figure S2, *GhWAK6* highly expressed in young roots; *GhWAK4*, *GhWAK5* and *GhWAK16* showed stem-specific expression; *GhWAK1*, *GhWAK3*, *GhWAK10*, *GhWAK17* and *GhWAK19* dramatically expressed in young leaves; *GhWAK2*, *GhWAK13*, *GhWAK18* and *GhWAK29* expressed in flower-specific manner; *GhWAK8*, *GhWAK9*, *GhWAK14*, *GhWAK15*, *GhWAK20*, *GhWAK21*, *GhWAK22*, *GhWAK23*, *GhWAK24*, *GhWAK27*, *GhWAK28* highly expressed in ovule-preferential manner. These results imply that *GhWAKs* expressed in tissue-specific manner in cotton.

### Transcriptome analysis of *GhWAK* genes during fiber cell development

**Fig. 5:**
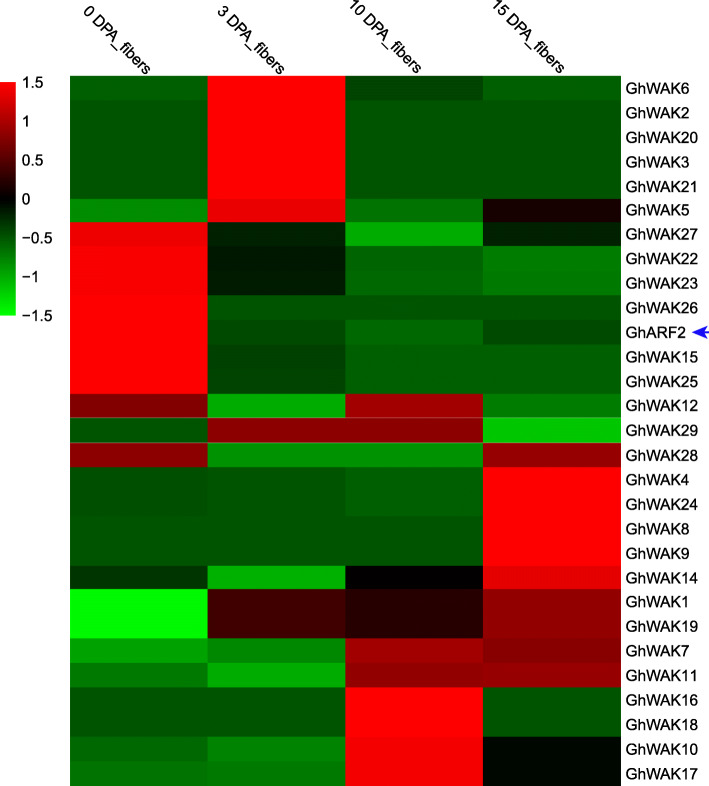
Heatmap of RNA-seq data of *GhWAK* gene expression levels during fiber cell development stages. The transcriptome data were normalized and visiualized by the pheatmap package with default parameters in R language. The colors from green to red indicate the expression levels from low to high, respectively. *GhARF2* is involved in fiber initiation development and used as a marker gene

To further explore whether *GhWAK* genes potentially contribute to fiber cell development, the expression profiles of each gene were investigated using transcriptome data from different developmental stages (0 DPA, 3 DPA, 10 DPA and 15 DPA). To better show the expression values, we introduced *GhARF2* gene (*GH_D12G2130*) [41], which highly expressed at the cotton fiber cell initiation stage, into the profile analysis of *GhWAK* genes. Our result showed that *GhARF2* highly expressed in 0 DPA, which is consistent with the published result [41]. Furthermore, we investigated the expression level of six *GhWAK* genes using qRT-PCR experiment. Our data showed that these *GhWAK* genes were up-regulated in 3 DPA samples (Additional file 1: Figure S3), which is similar to the results from the transcriptome data, suggesting that the transcriptome data is credible. Twenty-eight out of Twenty-nine *GhWAKs* showed expression values in cotton fiber development process. The *GhWAK*s highly expressed during the fiber cell development process (Fig. 5). *GhWAK2*, *3*, *5*, *6*, *12*, *15, 20*, *21, 22*, *23*, *25*, *26*, *27* and *GhARF2* showed high expression levels at fiber cell initiation stage (0 and 3 DPA). The other *GhWAK* members highly expressed at fiber cell elongation stage (10 and 15 DPA). *Arabidopsis* trichomes are organs similar to fiber cells in cotton [42].

**Fig. 6:**
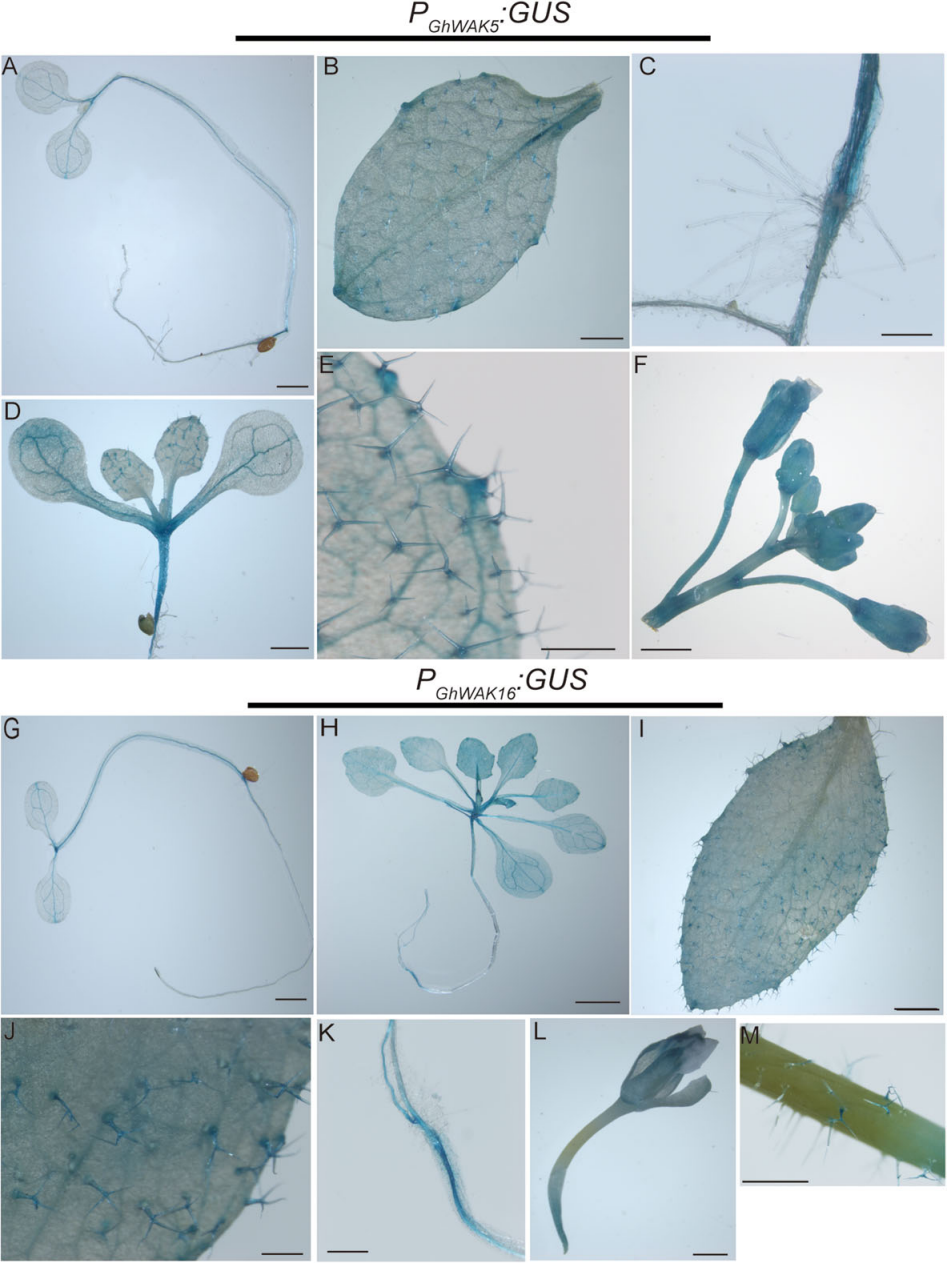
The phenotype of GUS staining of *P*_*GhWAK5*_*:GUS* and P_*GhWAK16*_:*GUS* transgenic *A. thaliana* plants. **a** and **g** Five-day-old transgenic seedlings. **d** eight-day-old transgenic plant. **h** 15-day-old transgenic plant. **c** and **k** Root. **f and l** flower. **m** stem. Scale bars = 2.5 mm in (**a**, **b**, **c**, **d**, **f**, **g**, **h**, **i**, **k**, **l**, **m**), 50 μm (**e**, **j**)

Atfer the initiation, fiber cells undergo the fast elongation stage (about 3 DPA to 10 DPA), in which fiber cells elongate about 2 mm per day. Therefore, we selected *GhWAK5* with the highest expression level at 3 DPA and *GhWAK16* with the peak value in 10 DPA fiber cells as the examples to further investigate the potential expression patterns of *GhWAKs* by GUS staining assays. The confused vectors *of P*_*GhWAK5*_*::GU*S and *P*_*GhWAK16*_*::GUS* were constructed and introduced into Arabidopsis. Various tissues at different developmental stages of the transgenic Arabidopsis were collected and stained by GUS solution. As shown in Fig. 6a-f, *GhWAK5* promoter-driven GUS expression was mainly observed in veins, trichomes, nodes, primary roots, hypocotyls and nodes. *GhWAK16* promoter-driven *GUS* gene expression mainly present in leaf veins and hypocotyls, trichomes, nodes, primary roots and nodes (Fig. 6g-m). These results suggest that *GhWAK5* and *GhWAK16* may contribute to fiber development because trichomes are the similar organs to cotton fibers.

### Analysis of *cis*-acting elements related to phytohormones in putative *GhWAK* promoter regions

Plant hormones play important roles in cotton fiber initiation, elongation, secondary cell wall deposition and maturation stages, and any slight change in its content will cause obvious changes in fiber qualities [35]. Cotton fibers are single cells and start from ovule epidermis. In order to investigate the potential regulatory mechanism of *GhWAK* genes, 2000 bp promoter sequences upstream from inition codons (ATG) were scanned in the PLACE database to obtain the *cis*-acting elements related to phytohormones. The results showed that fourteen *GhWAK* promoters harbor at least one salicylic acid (SA) related *cis*-acting elements (Additional file 2: Table S5); twenty, fourteen and ten *GhWAK*s contain jasmonic acid (JA), gibberellin (GA) and auxin related cis-acting elements, respectively, indicating that *GhWAK*s may be induced by various plant hormones.

### *GhWAK* genes were stimulated by phytohormones

**Fig. 7:**
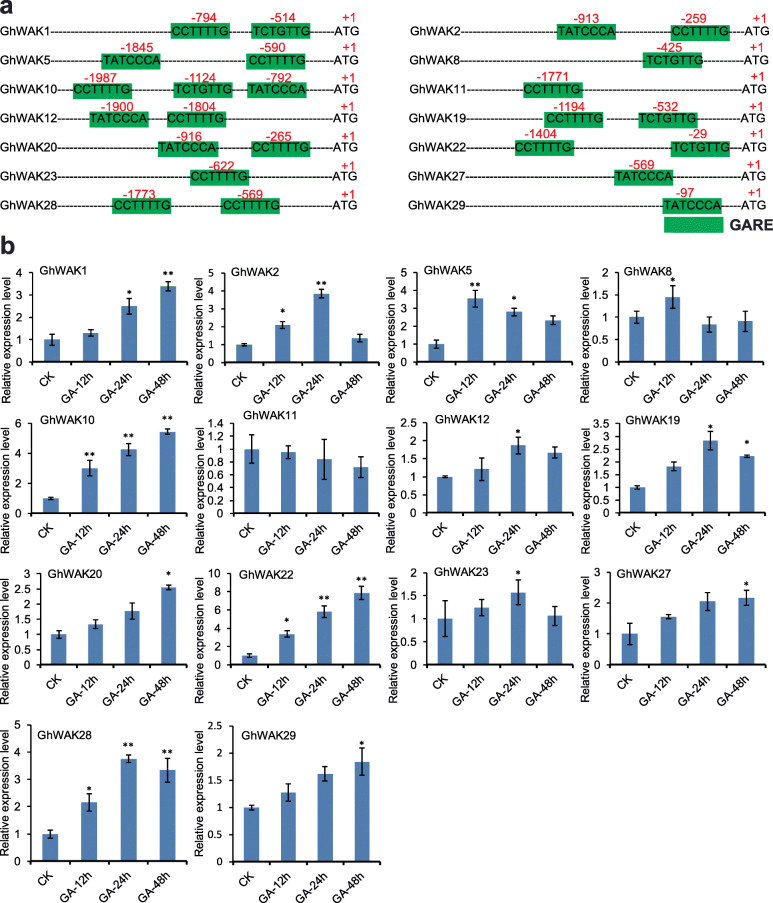
*GhWAK* gene transcription is induced by gibberellic acid (GA). **a** Identification of putative gibberellic acid response elements (GARE) in the promoter regions of the *GhWAK* gene family members. **b** GA-induced transcription in the *GhWAK* genes

Phytohormones, especially gibberellin and auxin, are reported to regulate cotton fibers cell development [43, 44]. In order to explore the relationship between *GhWAK* genes and gibberellin acid (GA), we analyzed the *cis*-elements in the *GhWAK* promoter regions. A number of gibberellin-responsive *cis*-elements (GAREs), including TCTGTTG, CCTTTTG and TATCCCA, were observed within *GhWAK* promoter regions (Fig. 7a). Fourteen out of 29 *GhWAK* genes possessed at least one *cis*-acting element involved in gibberellin-responsiveness. These results suggest that the expression level of *GhWAK* genes may be regulated by GA. To confirm this finding, expression analysis of *GhWAK* genes was carried out after treatment with GA. Our results showed that a total of 13 *GhWAK* genes were induced by GA treatment (Fig. 7b), except for *GhWAK11*.

**Fig. 8:**
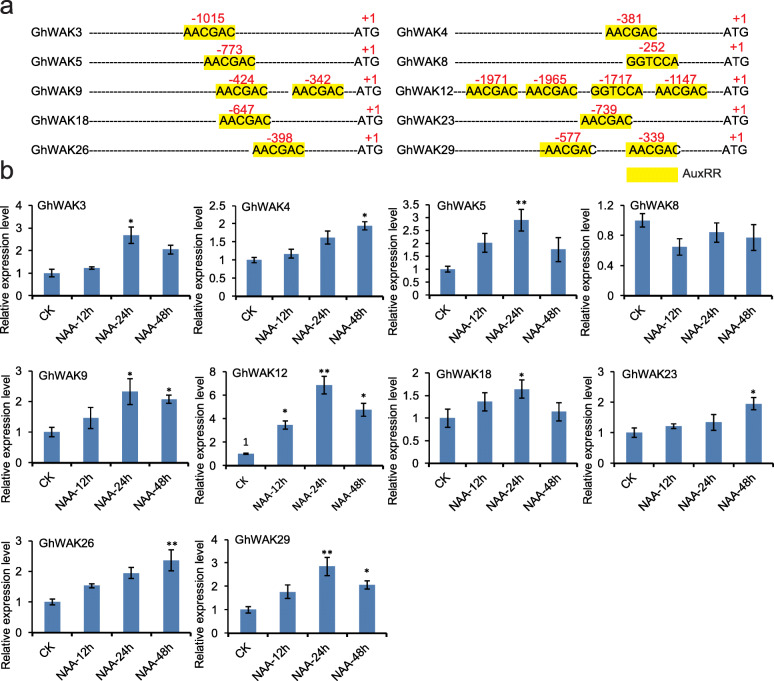
*GhWAK* gene transcription is induced by auxin (NAA). **a** Identification of putative *cis*-acting regulatory element involved in auxin responsiveness (AuxRRs) in the promoter regions of the *GhWAK* gene family members. **b** NAA-induced transcription in the *GhWAK* genes

Auxin is also reported to promote cotton fiber cell development [43]. In order to explore the relationship between *GhWAK* genes and auxin (IAA), we also searched the *cis*-acting regulatory element involved in auxin responsiveness. As a result, AuxRR-core (GGTCCAT and AACGAC), and two kinds of auxin-responsive *cis*-elements were successfully identified in the *GhWAKs* promoter regions. Our data showed that a total of ten *GhWAK* genes contained an auxin-responsive *cis*-element (Fig. 8a). The qRT-PCR analysis showed that the expression levels of nine *GhWAK* genes were significantly increased after treatment with auxin (Fig. 8b). These results suggest that *GhWAK* genes may be involved in phytohormone-mediated fiber cell development in cotton.

## Discussion

Wall-associated kinase (WAK), one of the receptor-like kinases (RLKs), is essential in signal transduction between the cell wall and the cytoplasm. WAKs are highly conservative and contains four typical domains (signal peptide, transmembrane, EGF and protein kinase). In *Arabidopsis*, there are five WAK proteins, which function directly in cellular cell elongation and cell wall formation [5]. However, the *WAK* gene family has not been investigated in *G. hirsutum*, which is famous for its high quality fiber [45].

### *GhWAK* family members amplified by gene duplication events

*G. hirsutum* is an allotetraploid species that is originated from hybridization of the female parent *G. arboreum* (AA, 2n = 2x = 26) and the male parent *G. raimondii* (DD, 2n = 2x = 26) 1–2 million years ago [30, 46]. In this study, 29 *GhWAKs*, 19 *GaWAKs* and 14 *GrWAKs* were identified from *G. hirsutum*, *G. arboretum* and *G. raimondii*, respectively. The number of *WAKs* in *G. hirsutum* was nearly six times that in *Arabidopsis*, indicating that gene expansion occurred in *WAK* gene family during the evolution of cotton genomes. Analysis of gene duplication events showed that *GrWAK* and *GaWAK* family members were mainly produced from tandem duplication and *GhWAK* family members were mainly produced from WGD. According to our divergence time analysis, the divergence time of *GhWAK*s, *GaWAKs* and *GrWAKs* were earlier than 6.97 MYA, which indicated that the gene duplication events occurred before the polyploidization event.

Previous phylogenetic analysis of WAK proteins among *Arabidopsis*, rice and *Populus* showed that WAK proteins form species-specific clusters [6, 47, 48], which indicates that WAK proteins are highly conserved during evolution. In this study, we identified a total of 29 *GhWAKs* in *G. hirsutum*, including 15 from the Dt subgenome and 14 genes from the At subgenome (Additional file 2: Table S1). Notably, 14 *GrWAKs* genes were found in the *G. raimondii* genome and 19 *GaWAKs* were found in the *G. arboreum* genome, suggesting that duplication events and gene loss occurred in the *GhWAK* gene family after the polyploidization in *Gossypium*, which further confirmed previous work showing that a large number of genes were lost in allotetraploid cotton [30]. Further chromosome localization analysis of *GhWAK*s shows that 29 *GhWAK*s are distributed unequally on 12 chromosomes and there are a number of genes clustered closely on one chromosome with high sequence similarity, which can prevent the loss of functions during evolution [49].

### Roles of *GhWAKs* in plant growth and hormone treatment

Plant cell wall is a complicated network structure that is involved in physiological processes such as plant cell growth and signal transduction. Studies in Arabidopsis, corn, rice and wheat showed that WAKs play functions in loosing cell wall to elongate cell and increasing cell wall strength to enhance pathogen resistance by promoting biosynthesis of cellulose and phytoalexin [9–12, 20–24]. Cotton fibers are single cells, and the cell length as well as cell wall strength determine fiber qualities [33, 34]. Considering the important functions of WAKs in plant cell walls, the promoters and expression patterns of *GhWAKs* were analyzed in *G. hirsutum*.

Salicylic acid (SA) and jasmonic acid (JA) are important signal molecules in plant pathgenon defense responses by promoting cell wall synthesis and integrity [50]. Previous studies have shown that *TtWAK2*, *OsWAK/Xa4*, and *ZmWAK-RLK1* can increase pathogen resistance by regulating cellulose synthesis to strength the cell wall [20, 24, 25]. In the promoter regions analysis, we found SA and JA responsive *cis*-elements in *GhWAK* promoter*s* (Additional file 2: Table S5). Furthermore, these *GhWAKs* highly expressed at 15 DPA, which was the secondary cell wall development stage, the important for fiber strength [51].

Both auxin and GA are phytohormones that promote cell elongation [52–54]. In this study, we identified auxin- and GA-responsive *cis*-elements harbored in most of the *GhWAK* promoter regions and these *GhWAK*s can also respond to GA and auxin. Coincidently, studies have shown that *WAK*s were involved in the regulation of cell elongation. Expression of an inducible antisense *AtWAK2* led to a 50% reduction in WAK protein levels, with a subsequent loss of cell elongation, and hence dwarf in *Arabidopsis* [8]. On the basis of *cis*-element analysis, 14 *GhWAKs* possessed at least one *cis*-acting element involved in gibberellin-responsiveness and 13 of them were significantly induced by gibberellin treatment. A total of ten *GhWAK*s were found to contain an auxin-responsive *cis*-element. Among them, nine *GhWAKs* were significantly induced after treatment with auxin (Figs. 7 and 8). GUS staining indicated that the *GhWAK5* and *GhWAK16* expressed in *Arabidopsis* trichomes, stems, roots and nodes. Also, previous studies demonstrated that trichomes are organs similar to cotton fibers [54]. Therefore, we speculated that *WAKs* may play functions in promoting the elongation of cotton cells.

## Conclusions

In the present study, we identified 29 *GhWAKs* from *G. hirsutum*. Phylogenetic analysis showed that cotton WAK proteins can be divided into five clades. The results of synteny and Ka/Ks analysis showed that the *GhWAKs* mainly originated from whole genome duplication (WGD) and were then mainly under purifying selection. Transcriptome data and qRT-PCR showed that 28 out of 29 *GhWAKs* highly expressed in cotton fibers. Both RNA-seq data and promoter GUS stainning analysis showed that *GhWAKs* expressed with tissue-specific manner. We also identified putative gibberellin (GA), auxin (IAA), jasmonate acid (JA) and salicylic acid (SA) response elements in the promoter regions. Furthermore, qRT-PCR data showed that the *GhWAK*s with transcripts significantly induced by GA and IAA. Fourteen *GhWAKs* possessed at least one *cis*-acting element involved in gibberellin-responsiveness and 13 of them were significantly indcued by gibberellin treatment. A total of ten *GhWAKs* were found to contain an auxin-responsive *cis*-element. Among them, nine *GhWAKs* were significantly induced after treatment with auxin. Phytohormones auxin and GA positively regulate cell elongation. Combining the high expression of *GhWAK* genes in fiber cells with the increased *GhWAK* transcripts induced by auxin and GA, we think that *GhWAK* genes might invovle in auxin- and GA-regulating cotton fiber growth.

## Methods

### Identification of WAKs in *G. hirsutum*, *G. arboreum* and *G. raimondii*

Genome sequences of *G. hirsutum* acc. TM-1 (ZJU_v2.1), *G. raimondii* (JGI_v2.1) and *G. arboreum* (CRI_v3.0) were downloaded from the CottonGen database (https://cottonfgd.org/about/download.html). The five AtWAKs of *A. thaliana* were downloaded from the *Arabidopsis* Information Resource (TAIR, Additional file 2: Table S6) [55] The WAKs of *G. hirsutum*, *G. raimondii* and *G. arboreum* were retrieved by BLASTp with threshold value 1e-5 [56]. Based on previous studies of WAK proteins in *Arabidopsis*, *Oryza sativa* and *Malus domestica* [3, 5, 27, 47], WAK proteins contain four typical conserved domains, signal peptide, 1–3 EGFs, a transmembrane domain and a protein kinase domain, therefore, all Blastp results were confirmed by identification of the four typical domains.

### Gene locations, structures and physicochemical properties analysis

The position information for all *GhWAK*s was obtained from the gene annotations in gene feature format (GFF) files were downloaded from the CottonGen database and visualized using MapChart [57]. The exon-intron structure of *GhWAK*s were analyzed according to the GFF and visualized by the online software Gene Structure Display Server Program (GSDS) [58].

The isoelectric point (pI) and molecular weight (MW) were predicted with the online software ExPASy [59]. The subcellular localization of GhWAK proteins was predicted using WoLF PSORT [60] online software. An instability index greater than 40 indicates that the in vivo half-life is less than 5 h and an index less than 40 indicate that the in vivo half-life is more than 16 h [61]. A grand average of hydropathicity (GRAVY) less than zero indicates that proteins are hydropathic [62].

### GhWAK protein alignment, phylogenetic analysis and conserved domain analysis

The multiple sequences of GhWAKs and five AtWAKs (AT1G21210.1, AT1G21230.1, AT1G21240.1, AT1G21250.1 and AT1G21270.1) were aligned using ClustalX 2.0 [63], and an unrooted phylogenetic tree was generated using the NJ method in MEGA 7.0 [64].

The conserved domains of GhWAKs were predicted using online software as described below. The signal peptides were scanned using SignalP-5.0 Server [65]; transmembrane domains were predicted using the Prediction of transmembrane helices in proteins (TMHMM Server v. 2.0) [66]; and the EGF and protein kinase domain were predicted using the ScanProsite tool [67].

### Duplication and Synteny analysis of *GhWAK* genes

The duplication types of *GhWAK*, *GrWAK* and *GaWAK* were classified using the MCScanX software with the default parameters [68], the synteny and collinearity were determined and analyzed using the Multiple Collinearity Scan toolkit (MCScanX) software [68]. The synteny relationships of *GhWAK*, *GaWAK* and *GrWAK* genes were visualized with Circos software [69]. The gene sequences of each paralogous gene pairs were used to calculate the synonym substitution (Ks) and non-synonymous substitution (Ka) by using the PAML package [70].

### Plant growth conditions and plant hormone treatments

*G. hirsutum* cultivar Xuzhou 142 was grown with a 16 h light and 8 h dark cycle at 30 °C under controlled climate conditions as previously reported [54, 71]. The fresh cotton fibers were separated from the ovules at 0, 3, 10 and 15 DPA and immediately frozen in liquid nitrogen. Experiments of phytohormone treatment were performed with 30 cotton ovules at 1 day post anthesis (1 DPA) and each treatment had three biological replicates [72]. The 1 DPA ovules were carefully dissected, sterilized and cultured on liquid medium [71, 72], containing 1 μM auxin 1-Naphthylacetic acid (NAA, Sigma), 1 μM gibberellin acid (GA_3_, Sigma), or the treatment without any phytohormone as the control (CK), respectively. After the treatments, the ovules were collected and immediately frozen in liquid nitrogen, and stored at − 80 °C for qRT-PCR experiments.

### Identification of plant growth regulator-related *cis*-elements

The upstream 2000 bp genomic DNA sequences of the initiation codon (ATG) were proposed as promoter regions for *GhWAK*s [72, 73]. The *cis*-elements of *GhWAK* promoter regions were detected using the plant *cis*-acting regulatory element (Plant CARE) database with the default parameters [74]. Phytohormones-related *cis*-elements were summarized and tabulated in Additional file 2: Table S5.

### GUS staining analysis of *GhWAK5* promoter

The putative genomic promoter sequences of *GhWAK5* and *GhWAK16* were cloned from Xuzhou 142, respectively. Then, the cloned putative promoters were assembled into the vector pCAMBIA1300 by In-Fusion® HD Cloning Kit (Vazyme Biotech, China) to drive the *GUS* gene expression. The fused vector was introduced into Arabidopsis using transformation method as previous work [75]. All the primers and cutting-sites used in vector construction are listed in Additional file 2: Table S7.

For GUS staining, various tissues at different developmental stages of the transgenic Arabidopsis were collected, stained with GUS staining solution according to the instruction and then washed with 70% ethanol for several times to remove background [76]. Stained samples were observed with DMRX microscope (Leica, Germany) and photographed by digital camera (Nikon, Japan).

### RNA isolation and expression profiling analysis

The expression levels of *GhWAKs* in different tissues (stem, root, leaf and ovules) and fibers at various developmental stages (0 DPA, 3 DPA, 10 DPA and 15 DPA) were obtained from previously reported transcriptome data [77]. The datas were available in the National Center for Biotechnology Information (NCBI) under accession number SRA180756. The expression analysis of each gene was conducted and normalized using the fragments per kilobase of transcript per million mapped reads (FPKM) method. To show *GhWAK* expression patterns during fiber development, an auxin responsive factor gene (*GhARF2*, *GH_D12G2130)* was used as a marker gene, which is one of the key regulators of cotton fiber initiation [41]. The transcriptome data were normalized and visualized using the pheatmap package with default parameters in an R environment (https://stackoverflow.com/questions/33292067/pheatmap-annotation-colors-and-border).

Total RNA from ovules treated with plant hormone as described above was isolated using the PureLink™ RNA mini kit (Invitrogen, Lot no. 1687455) and the cDNA was reverse-transcribed from 1.0 μg total RNA using PrimeScript RT Regent kit (Takara, Japan). OLIGO 7 was used to design gene-specific primers [78] for quantitative real-time PCR (qRT-PCR) (Additional file 2: Table S7).

The qRT-PCR assays were performed using a Bio-RadReal Time PCR detection system (Bio-Rad CFX96Touch, USA). The SYBR Green qRT-PCR reactions contained 10 μl SYBR® Premix Ex Taq™ II (Takara, Japan), 0.5 μl of 10 μM primers, and 2 ng cDNA template. The final volume was adjusted to 20 μl with ddH_2_O. The PCR cycling conditions were 95 °C for 30 s, followed by 40 cycles of 95 °C for 5 s and 60 °C for 30 s. The cotton ubiquitin gene *GhUBQ7* was used as the internal control for normalization of gene expression in each qRT-PCR experiment [79]. The experiment was performed with three biological replicates and each biological replicate was performed with three technical replicates. The expression levels were compared with the control treatments and relative gene expression was calculated using the 2^−ΔΔCt^ method [80]. SPSS 16.0 software was used for one-way statistical variance to analyze relative expression levels [81].

## Supplementary Information

**Additional file 1: Figure S1.** Synteny comparison of WAK regions from the homeologous in three cotton genomes. Black and orange ovals indicate *WAK* genes from dispersed and proximal duplication, respectively. The red lines indicate *WAK* gene from WGD. Green dots indicate *WAK* gene from tandem duplication. The blue lines indicate orthologous gene pairs. **Figure S2.** Heatmap of RNA-seq data of *GhWAK* gene expression levels in five different tissues of *G. hirsutum* (Xuzhou 142). The transcriptome data were normalized and visualized by the pheatmap package in R language. The colorful bars from green to red indicate the expression levels from low to high, respectively. **Figure S3.** Expression profile of six *GhWAK* genes during fiber cell development stages.

**Additional file 2: Table S1.** Physicochemical parameters of 14 *GrWAK* and 19 *GaWAK* genes in *G. raimondii* and *G. arboreum* genomes. **Table S2.** Analysis of duplication events of *GhWAK*, *GaWAK* and *GrWAK* in *G. hirsutum*, *G. arboreum* and *G. raimondii*, respectively. **Table S3.** Orthologous and paralogous relationships among *WAK* genes in *G. hirsutum*, *G. arboreum* and *G. raimondii.* **Table S4.** Ka/Ks analysis and divergence times of duplicated *GhWAK* gene pairs of *G. hirsutum*, *G.raimondii* and *G. arboreum*. **Table S5.** *cis*-elements related to plant growth regulators in the promoters of *GhWAK* genes. **Table S6.** Physicochemical parameters of five *AtWAK* genes in the *A. thaliana* genome. **Table S7.** *GhWAK* gene primer pairs used for qRT-PCR and GUS staining.

## Data Availability

The genome sequences of *G. hirsutum* acc. TM-1 (ZJU_v2.1), *G. raimondii* (JGI_v2.1) and *G. arboreum* (CRI_v3.0) are available in the CottonGen website (https://cottonfgd.org/about/download.html). The Arabidopsis genome was available from the TAIR 10 (https://www.arabidopsis.org/). The transcriptome data of *G. hirsutum* is available from NCBI Sequence Read Archive (SRA) with the accession number SRA180756.

## Abbreviations

RLK: Receptor like kinase
ECD: Extracellular domain (ECD
WAK: Wall-associated kinase
PERK: Proline rich extensine-like receptor kinases
EGF: Epidermal growth factor-like
ELISA: Enzyme-linked immunosorbent assay
GA: Gibberellin
IAA: Auxin
DCA: Deoxycholic acid
SA: Salicylic acid
DPA: Day post-anthesis
NJ: Neighbor-joining
WGD: Whole genome duplication
MYA: Million years ago
Ka: Non-synonymous
Ks: Synonymous
GAREs: Gibberellin-responsive *cis*-elements
GRAVY: Grand average of hydropathicity
NAA: 1-Naphthylacetic acid
JA: Jasmonic acid
