# Supplementary figures and images for: Genome-wide characterization of the WAK gene family and expression analysis under plant hormone treatment in cotton
Source: BMC Genomics. 2021 Jan 28;22:85. doi: 10.1186/s12864-021-07378-8 (PMC7842020; doi:10.1186/s12864-021-07378-8)

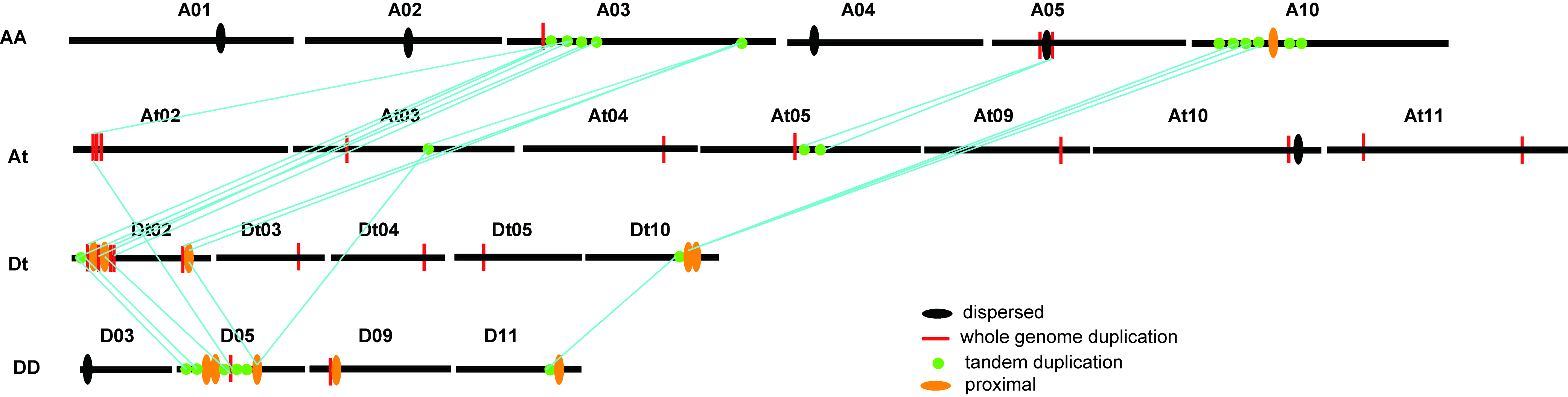

Supplement: Supplementary file 1 — Additional file 1: Figure S1. Synteny comparison of WAK regions from the homeologous in three cotton genomes. Black and orange ovals indicate WAK genes from dispersed and proximal duplication, respectively. The red lines indicate WAK gene from WGD. Green dots indicate WAK gene from tandem duplication. The blue lines indicate orthologous gene pairs. Figure S2. Heatmap of RNA-seq data of GhWAK gene expression levels in five different tissues of G. hirsutum (Xuzhou 142). The transcriptome data were normalized and visualized by the pheatmap package in R language. The colorful bars from green to red indicate the expression levels from low to high, respectively. Figure S3. Expression profile of six GhWAK genes during fiber cell development stages. [file 12864_2021_7378_MOESM1_ESM.zip › Additional file 1 Figure S1.tif]

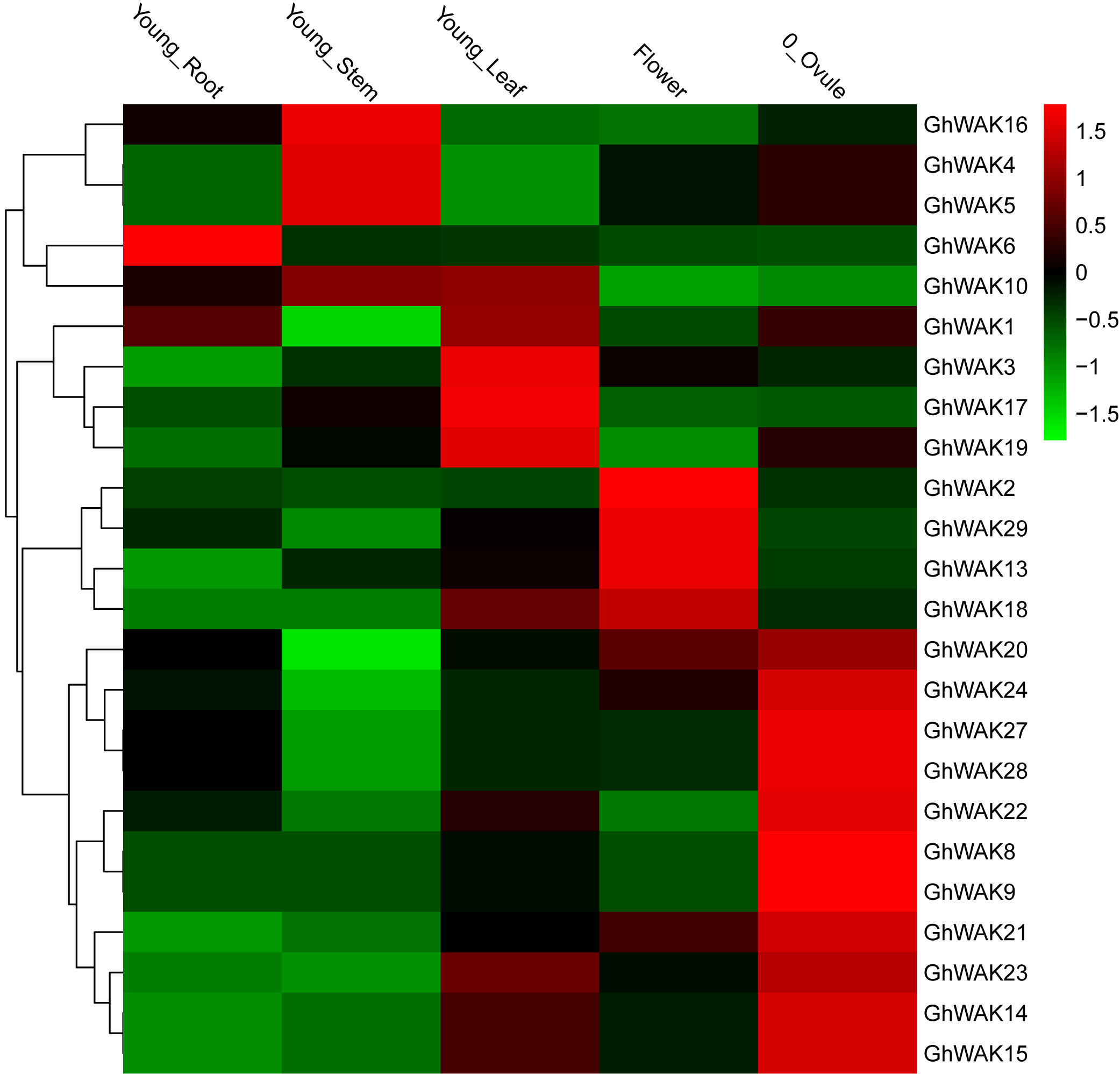

Supplement: Supplementary file 1 — Additional file 1: Figure S1. Synteny comparison of WAK regions from the homeologous in three cotton genomes. Black and orange ovals indicate WAK genes from dispersed and proximal duplication, respectively. The red lines indicate WAK gene from WGD. Green dots indicate WAK gene from tandem duplication. The blue lines indicate orthologous gene pairs. Figure S2. Heatmap of RNA-seq data of GhWAK gene expression levels in five different tissues of G. hirsutum (Xuzhou 142). The transcriptome data were normalized and visualized by the pheatmap package in R language. The colorful bars from green to red indicate the expression levels from low to high, respectively. Figure S3. Expression profile of six GhWAK genes during fiber cell development stages. [file 12864_2021_7378_MOESM1_ESM.zip › Additional file 1 Figure S2-Tissues-pheatmap.tif]

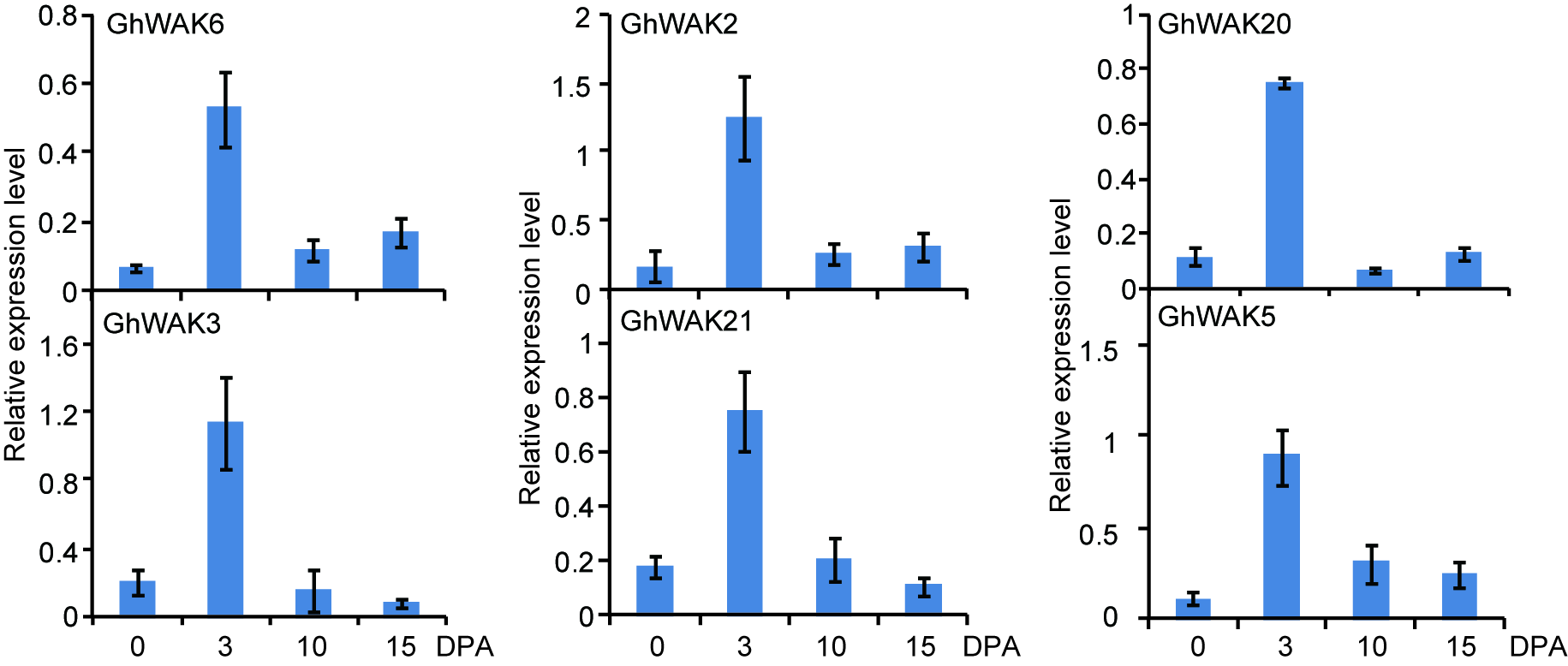

Supplement: Supplementary file 1 — Additional file 1: Figure S1. Synteny comparison of WAK regions from the homeologous in three cotton genomes. Black and orange ovals indicate WAK genes from dispersed and proximal duplication, respectively. The red lines indicate WAK gene from WGD. Green dots indicate WAK gene from tandem duplication. The blue lines indicate orthologous gene pairs. Figure S2. Heatmap of RNA-seq data of GhWAK gene expression levels in five different tissues of G. hirsutum (Xuzhou 142). The transcriptome data were normalized and visualized by the pheatmap package in R language. The colorful bars from green to red indicate the expression levels from low to high, respectively. Figure S3. Expression profile of six GhWAK genes during fiber cell development stages. [file 12864_2021_7378_MOESM1_ESM.zip › Additional file 1 Figure S3.tif]
